# Confidence Predictions Affect Performance Confidence and Neural Preparation in Perceptual Decision Making

**DOI:** 10.1038/s41598-019-40681-9

**Published:** 2019-03-11

**Authors:** Annika Boldt, Anne-Marike Schiffer, Florian Waszak, Nick Yeung

**Affiliations:** 1 Department of Experimental Psychology, University of Oxford, Oxford, OX2 6GG UK; 2 Department of Psychology, University of Cambridge, Cambridge, CB2 3EB UK; 3 Institute of Cognitive Neuroscience, University College London, London, WC1N 3AZ UK; 4 CNRS (Integrative Neuroscience and Cognition Center, UMR 8002), Paris, France; 5 Université Paris Descartes, Sorbonne Paris Cité, Paris, France

## Abstract

Decisions are usually accompanied by a feeling of being wrong or right – a subjective confidence estimate. But what information is this confidence estimate based on, and what is confidence used for? To answer these questions, research has largely focused on confidence regarding current or past decisions, for example identifying how characteristics of the stimulus affect confidence, how confidence can be used as an internally generated feedback signal, and how communicating confidence can affect group decisions. Here, we report two studies which implemented a novel metacognitive measure: predictions of confidence for future perceptual decisions. Using computational modeling of behaviour and EEG, we established that experience-based confidence predictions are one source of information that affects how confident we are in future decision-making, and that learned confidence-expectations affect neural preparation for future decisions. Results from both studies show that participants develop precise confidence predictions informed by past confidence experience. Notably, our results also show that confidence predictions affect performance confidence rated after a decision is made; this finding supports the proposal that confidence judgments are based on multiple sources of information, including expectations. We found strong support for this link in neural correlates of stimulus preparation and processing. EEG measures of preparatory neural activity (contingent negative variation; CNV) and evidence accumulation (centro-parietal positivity; CPP) show that predicted confidence affects neural preparation for stimulus processing, supporting the proposal that one purpose of confidence judgments may be to learn about performance for future encounters and prepare accordingly.

## Introduction

The decisions we make are usually accompanied by a feeling of whether the decision was wrong or right. This pertains to a wide range of decisions, such as selection of an action, placement of a bet, or perceptual decisions such as whether we can cross the road safely or will be hit by a car. This performance estimate, called a *confidence judgment*, is taken to reflect the probability of being correct^1^. There is ongoing debate about *how* – and based on what information – confidence judgments are formed (e.g.^2^). Further, the purpose of this internal judgment is likewise a matter of debate^3,4^. The present study tests the idea that experience-based confidence predictions affect how confident we are in future decision-making and that one purpose of confidence is to learn about performance to prepare for future encounters of a task.

Performance confidence is influenced by physical properties of the stimulus that a decision is based on. For example, the quality of evidence favoring a decision (which impacts accuracy and reaction times) has been shown to affect confidence, thus establishing an internally generated feedback-signal^5–9^. However, many everyday examples suggest that confidence judgments result from the integration of multiple cues, beyond physical characteristics of a stimulus: A classic example of a perceptual decision is that of a trained oncologist’s ability to discriminate between cancerous and healthy tissue on X-rays. Multiple cues such as familiarity with the decision and past experience of failure and success will likely contribute to her confidence. In other words, experience of high or low confidence in the past may lead to specific predictions of confidence in the present decision. Therefore, we hypothesize that humans not only experience confidence in current decisions, but also learn about such confidence, leading to confidence predictions for future decisions of the same type. We propose that repeated experience (*performance confidence*) leads to the formation of discrete expectations (*predicted confidence*). Conversely, we propose that predicted confidence is one of the cues affecting performance confidence.

The concept of predicted confidence has mostly been explored outside the field of perception in research on voluntary choices and investments into cognitive tasks (^10,11^ for review), and also in memory research, here, addressing whether confidence in being able to recall an item later (*judgements of learning*; JOLs) affects how much time we allocate to studying this item^12^. A recent study by Fleming and colleagues^13^ yielded several interesting findings, demonstrating that participants are capable of forming accurate global prospective confidence judgements regarding an upcoming perceptual decision, and that such predictions are largely based on their accuracy over several previous trials. However, in their paradigm, accuracy across the entire experiment was fixed using a staircase procedure. The question therefore remains whether people are capable of tracking highly specific confidence predictions. More fundamentally, it remains unclear whether predicted confidence affects performance and associated confidence during perceptual decision-making processes, or whether these aspects of perceptual decisions rely purely on the quality of the evidence on which the decision is based (and is not informed by expectations).

The current study investigates two main questions. First, we tested whether human observers acquire stimulus-type specific confidence predictions based on experienced confidence in perceptual decision making. Further, we hypothesized that confidence predictions would be flexibly updated when changes in task-contingencies led to mismatches between predicted and performance confidence. We used EEG to measure whether neural signatures of stimulus preparation and stimulus processing would show a modulation by (predicted) confidence, which would furthermore suggest that predicted confidence is used to guide how people approach upcoming decisions. Second, we hypothesized a bi-directional link between prediction and experience, meaning that confidence predictions would not only be influenced by, but also modulate performance confidence. Establishing an influence of confidence predictions on performance confidence would support the notion that confidence estimates integrate multiple cues, extending beyond the two best established factors: the stimulus’ physical properties and - partly as a result of the latter - the reaction time of the perceptual decision (cf^7^.).

Crucially, these ideas do not imply that predicted and performance confidence represent readouts of two entirely separable signals. Instead, this multiple cue account is closely related to a Bayesian framework of human cognition^14,15^, which proposes that continuous and flexible updating of beliefs based on both existing beliefs (priors) and newly incoming information is key to efficient successful behavior^16–18^. In this model, predicted confidence acts as a prior for performance confidence and the continuous update of prior confidence allows participants to form accurate beliefs about their own performance. What people report after the decision, termed performance confidence in the present study, translates into a posterior that integrates the prior with components of the decision itself. To summarize, we hypothesize that experience-based confidence predictions have an effect on stimulus processing and performance confidence. Moreover, mismatches between prediction and experienced confidence are expected to promote an update, so that future predictions are more precise. To measure these updates irrespective of task-performance, we implemented a task in which there are no known strategy changes that affect performance.

Our task consisted of a two-alternative forced-choice color judgment. Visual cues preceded the target stimulus and were matched to specific stimulus configurations known to elicit four different levels of performance confidence^7,19^. These four different confidence levels have been shown to dissociate partly from accuracy, allowing us to test whether subjective performance confidence, rather than objective accuracy, drives learning of confidence predictions. We expected the cues to become associated with the specific performance-confidence levels, as expressed in self-reported predicted confidence towards the cue. We further employed EEG to measure whether confidence predictions affect neural processing: confidence predictions were hypothesized to scale with the CNV component, a negative going slow-wave potential preceding the onset of the task-relevant stimulus, which is associated with the readiness to process a stimulus^20^. We expected that higher confidence predictions would lead to a larger CNV component, signifying the readiness to respond quickly under conditions of increased confidence.

We further expected that a neural marker of perceptual decision making - the centro-parietal positivity (CPP) component - would show differences in amplitude between the four different stimulus categories. The CPP is associated with accumulation of evidence in favor of one of two options in a decision, and peaks at the time at which the decision is made^21,22^. CPP built-up rate (slope) and amplitude have been shown to be related to reaction times^21,22^ and to be affected by internal influences on stimulus processing, such as attention^22^. Our experiment allowed us to test whether the behavioral differences between conditions, particularly differences in confidence, would be associated with differences in CPP amplitude.

## Results

### Experiment 1

**Figure 1:**
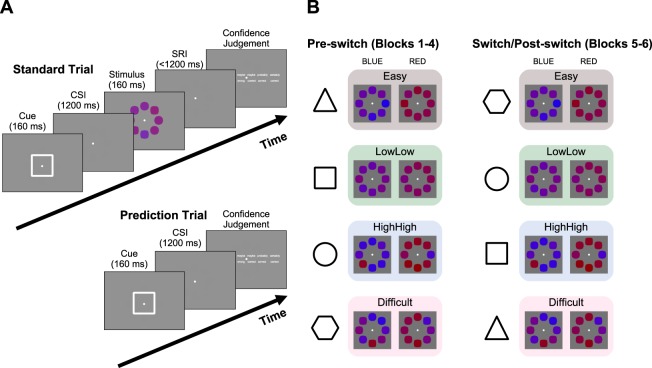
(**A**) The perceptual decision task consisted of two trial types. During Standard Trials, participants first viewed a visual (shape) cue. They then had to indicate whether an array of eight colored shapes was on average more red or more blue by pressing the left or right response key. The colored shapes were spaced regularly around a fixation point (radius 2.8″ visual arc). After making their response, the confidence scale was presented on screen and participants were given unlimited time to choose how confident they were that their last response was correct. During Prediction Trials, participants were also presented with the cue but instead of viewing the color stimulus, they were asked to rate how confident they were that they would have given a correct response, had they been presented with a stimulus. CSI: cue-stimulus interval; RSI: stimulus- response interval. (**B**) Sample stimuli, showing the four difficulty conditions in the 2 (mean) × 2 (variance) × 2 (color) design. The color stimuli were preceded by visual shapes that acted as predictive cues. The cue-condition contingency was reversed after Block 4 in Experiment 2. Color values are made more extreme for illustrative purposes.

The core task in both experiments consisted of an established paradigm in which participants had to indicate whether an array of 8 red, purple, and blue shapes was on average more red or more blue^7,19,23–25^ (Fig. 1). Subsequently, they had to indicate how certain they were that their response was correct or incorrect^7,19^. A schematic representation of the task structure together with the four difficulty conditions is given in Fig. 1, Panel A, referred to as the Standard Trial.

Stimuli varied along two dimensions: First, they differed in terms of the mean evidence in the distribution of colored shapes that formed the array. Decreasing the mean (i.e., using colors that were, on average, purple hues rather than clear red or blue) made the task more difficult. Second, they differed in terms of the variance in the distribution of colors. Increasing the variance (i.e., using colors that were a heterogeneous mix of reds, blues and purples rather than a homogeneous hue) made the task more difficult. Four conditions were created by factorial crossing of these two stimulus dimensions: An Easy condition had a high mean (on average the color was clearly red or clearly blue) and low stimulus variance (all shapes exhibited this difference). Conversely, the Difficult condition had a low stimulus mean (the average color was “purplish red” or “purplish blue”) and variance was high (some shapes were red and others blue). Crucially, the two remaining conditions were both of Medium difficulty, but with this difficulty reflecting different stimulus characteristics. The first medium condition was the low mean, low variance condition - the average color was “purplish red” or “purplish blue”, but the shapes were relatively homogeneous in color. The second Medium condition was the high mean, high variance condition - the average color was distant from the category boundary but the shapes differed widely from one another in hue (e.g., including clearly red and clearly blue patches in the same array). Performance was matched between these two medium conditions using the staircase procedure described in the Methods. Because performance (matched) and confidence (which differed, see below) dissociate in medium conditions, these provide an ideal test for the hypothesis that predicted confidence would follow confidence judgments, rather than objective accuracy.

The novel feature of the task compared to previous work^7,19,23–25^ was the introduction of predictive cues that preceded the color array (target stimulus) by 1200 ms. We used 5 different cues, consisting of familiar shapes (square, triangle, circle, pentagon, diamond). Four of the cues were matched to a specific condition (see below), with each cue always preceding the same condition (randomized across participants). The fifth cue preceded each of the four conditions equally often (neutral cue, hereafter). Participants were told that the cues might relate to how they feel about a trial, but not told explicitly about the systematic relationship to the conditions, nor that each stimulus could be categorized as falling into one of four conditions.

Following each perceptual decision, participants had to indicate their confidence in this decision (*performance confidence*) on a six-point scale (“certainly wrong”, “probably wrong”, “maybe wrong”, “maybe correct”, “probably correct”, “certainly correct”).

#### Performance Measures

**Figure 2:**
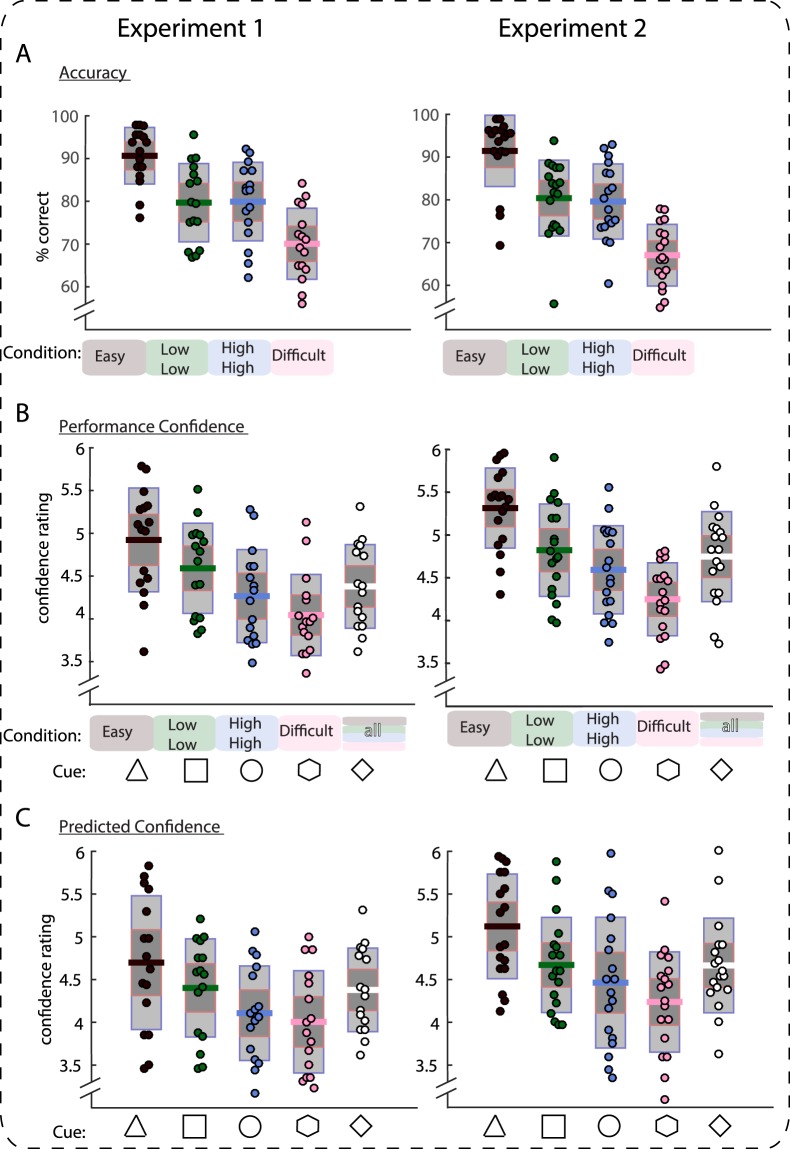
Accuracy (top), performance confidence (middle) and predicted confidence (bottom) in Experiment 1 (left) and Experiment 2 (right). Performance differs between easy and medium, easy and difficult, and medium and difficult trials, but not reliably between the two medium conditions (low low, high high). Confidence (both related to performance on the last trial and predictions concerning the next stimulus) differs between medium conditions, with the low low condition leading to higher confidence estimates than the high high condition. Single dots show single-subject data, bars display mean of the distribution (solid line), 95% confidence interval (dark grey shaded area), and 1 standard deviation (light grey shaded area). The data presented for Experiment 1 comprises all six experimental blocks, whereas the data presented for Experiment 2 comprises only the first four of six experimental blocks (those prior to the switch in the cue-condition contingencies).

Effects of condition were tested in repeated-measures ANOVAs, and planned paired-sample *t*-tests between conditions (two-tailed *α*-level = 0.05). Participants showed graded accuracy of responses between conditions (Fig. 2, Panel A). As expected, participants reached the highest percentage of correct responses in the easy condition (90.7%), and performed worst in the difficult condition (70.1%). Further, the medium conditions were well matched, with accuracy in the low mean, low variance condition at 79.7% and in the high mean, high variance condition at 79.9%. In a univariate ANOVA with CONDITION (4-levels) as independent variable, this factor had a significant effect (*F*(3,45) = 74.9, *p* < 0.001, *η*_*p*_^2^ = 0.83, 95% CI [0.81, 0.88]). Four paired-samples *t*-tests between the easy condition and each medium condition respectively as well as between the difficult condition and each medium condition showed significant differences (all *p* < 0.001, all *t*(15) > 7.1, all *r equivalent* > 0.79, see Supplemental Table 1 for details). In contrast, the paired-samples *t*-test between mean accuracy scores in the medium conditions showed no significant difference (*t* < 1); we thus find that objective accuracy was well-matched between the two medium conditions. The Bayes Factor in favor of this Null hypothesis of equality was *BF*_01_ = 3.87.

Reaction times (RTs) of correct responses showed the same overall pattern, with the fastest RTs in the easy condition (726 ms) and the slowest mean RTs in the most difficult condition (824 ms), again replicating previous findings. RTs averaged 770 ms in the low mean, low variance condition and 809 ms in the high mean, high variance condition. Entering RTs into a univariate ANOVA with CONDITION (4-levels) as independent variable, we found this factor to have a significant effect on RTs (*F*(3,45) = 15.9 *p* < 0.001, *η*_*p*_^2^ = 0.51, 95% CI [0.39, 0.66]). In contrast to our previous findings^7^, however, RTs in medium conditions differed significantly in a paired-samples *t*-test (*t*(15) = 2.4, *p* = 0.03, *r equivalent* = 0.41, 95% CI [0.14, 0.64]) with slower responses in the high mean, high variance condition than the low mean, low variance condition.

Participants were most efficient in the easy condition (779.61 IES; inverse efficiency score^26^ calculated as median correct RT divided by accuracy), and least efficient in the difficult condition (1160.25 IES). Efficiency in medium conditions fell between efficiency scores in the easy and difficult conditions (low mean, low variance: 946.46 IES, high mean, high variance: 993.84 IES). The univariate ANOVA again showed that CONDITION had a significant effect on performance (*F*(3,45) = 41.1, *p* < 0.001, *η*_*p*_^2^ = 0.73, 95% CI [0.69, 0.82]). Efficiency between medium conditions did not differ significantly (*t*(15) = 1.3, *p* = 0.21, *r equivalent* = 0.23, 95% CI [−0.09, 0.53]). The Bayes Factor in favour of this Null hypothesis of equality was *BF*_*01*_ = 1.87.

#### Performance Confidence

The first of the two main analyses aimed to establish the hypothesized graded effect of conditions on performance confidence, with a significant difference between medium conditions. The results support this hypothesis, with a main effect of CONDITION in a univariate ANOVA (*F*(3,45) = 20.3, *p* < 0.001, *η*_*p*_^2^ = 0.58, 95% CI [0.45, 0.72]), and a significant difference of average performance confidence between the two medium conditions in a planned pairwise contrast (*t*(15) = 2.4, *p* = 0.03, *r equivalent* = 0.40, 95% CI [0.13, 0.64], Fig. 2). As predicted, performance confidence was lower in the high mean, high variance condition than the low mean, low variance condition^7,19^ (Fig. 2, Panel B). Confidence further differed predictably between the easy condition and both medium conditions as well as between the difficult condition and both medium conditions (all *t*s > 3.2, all *p*s < 0.006, all *r equivalent* > 0.50, Supplementary Table 1 for details). See Supplementary Information an analysis of error awareness.

#### Predicted Confidence

In addition to the standard trials, we introduced confidence prediction trials, which made use of the predictive cues in our novel version of the task. These confidence prediction trials started exactly as the standard trials with the presentation of a predictive cue. However, following the 1200 ms delay, participants were presented with a confidence-judgment scale instead of the stimulus array. Their task was to estimate: “how well [they] think [they] would do on a trial that is preceded by the symbol [they] have just seen”. The scale was identical to the 6-point scale for performance confidence judgments in standard trials. Following this confidence rating (*predicted confidence*), an entirely new trial began following the ITI (of 1 second).

We found that predicted confidence fully matched with performance confidence. There was a strong influence of CONDITION on predicted confidence (*F*(3,45) = 10.5, *p* < 0.001, *η*_*p*_^2^ = 0.41, 95% CI [0.23, 0.60]) in the univariate ANOVA (Fig. 2, Panel C). Of particular interest, we observed a significant difference between predicted confidence in the medium conditions (*t*(15) = 2.9, *p* = 0.01, *r equivalent* = 0.46, 95% CI [0.21, 0.68]), with confidence again being lower in the high mean, high variance condition than the low mean, low variance condition. As expected, confidence further differed significantly between the easy condition and both medium conditions as well as between the difficult condition and both medium conditions (*p*s < 0.049, *t*s > 2.1, all *r equivalent* > 0.37, Supplementary Table 1 for details).

Thus, participants learnt to accurately predict their confidence across conditions, even for distinctions as subtle as the performance confidence difference across two conditions that were objectively well-matched in objective accuracy and overall performance efficiency. As such, these results provide strong evidence that human observers develop stimulus-category specific confidence predictions in decision making. Experiment 2 built on this conclusion to investigate the neural correlates of these confidence predictions, and their impact on performance confidence.

### Experiment 2

#### Behavior

Experiment 2 contained the same types of trials - standard trials and prediction trials - as Experiment 1. All variables of stimulus presentation and response mode were also kept constant, and the staircase procedure was identical. The experiments differed only with regard to the EEG recordings and one crucial element of the design: In Experiment 1, the cue-condition contingencies were stable for all 6 experimental blocks whereas in Experiment 2, the cue-condition contingencies reversed after the fourth of 6 blocks. Specifically, the cue that preceded trials of the easy condition in Blocks 1–4 preceded trials of the difficult condition in Blocks 5 and 6, and vice versa. A corresponding switch in cue-condition contingencies after 4 blocks was applied to the two medium conditions. The neutral cue remained neutral (non-predictive) throughout all 6 blocks.

This variation of the setup allowed us to test for two important predictions concerning the influence of confidence predictions on behavior: First, switching of the cues provided us with a sensitive measure of the influence of confidence predictions on performance confidence. We expected to find altered performance confidence judgments in the block immediately following the switch (Block 5). Specifically we expected that performance confidence would integrate predicted confidence (based on previous cue-confidence contingencies) and therefore be biased towards the mean. In detail, we predicted that cues previously associated with low confidence would decrease performance confidence after easy and low mean, low variance trials; therefore, confidence in these trials should be lower in the post-switch block (Block 5) than in the pre-switch block (Block 4). Conversely, we expected performance confidence on difficult and high mean, high variance trials to be higher in the post-switch block (Block 5) than in the pre-switch block (Block 4). The rationale for this prediction was that participants’ expectation to perform well in the task (driven by a cue so far associated with highly confident trials) would contribute to their performance confidence judgment and bias it towards higher confidence. The second aspect of this design is that it allows us to test the degree to which feedback-free learning of confidence associations is dynamic and rapidly modified by experience. We hypothesized that predicted confidence would show gradual adaption to the new cue-condition contingencies: We hence expected confidence predictions to be realigned with confidence judgments towards the end of the experiment (Block 6), but not immediately after the switch of cue-condition contingencies (Block 5).

##### Performance Measures

As in Experiment 1, performance measures were accuracy, RT, and efficiency. We applied the same measures as in Experiment 1: univariate ANOVAs of CONDITION, and planned *t*-tests (with α = 0.05). Further, because Experiment 2 included the crucial design element of a switch of cue-condition contingencies, we also introduced SWITCH (pre/switch) as a factor in the analysis. In these analyses, we directly compare two blocks prior to, during, or after the switch. For all analyses, we explicitly state which blocks were analyzed.

Analysis of RTs and accuracy in the four blocks before the switch of cue-contingencies replicated the main effects established in Experiment 1. The 4-level univariate ANOVAs showed significant effects of CONDITION both for accuracy (*F*(3,51) = 87.4, *p* < 0.001, *η*_*p*_^2^ = 0.84, 95% CI [0.79, 0.90]; Fig. 2) and for correct RTs (*F*(3,51) = 40.8, *p* < 0.001, *η*_*p*_^2^ = 0.71, 95% CI [0.62, 0.81]). We established again that accuracy in the easy and difficult conditions differed significantly from both medium conditions, in four respective paired *t*-tests (all *p*s < 0.001, all *t*s > 6.9, all *r equivalent* > 0.76, Supplemental Table 1 for details). As previously shown, there was no significant difference between medium conditions in percent correct responses (*t* < 1). The Bayes Factor in favor of this Null hypothesis of equality was *BF*_01_ = 3.58. Participants were again slower in the high mean, high variance condition than in the low mean, low variance condition (*t*(17) = 4.0, *p* = 0.001, *r equivalent* = 0.56, 95% CI [0.36, 0.75]).

These RT differences have predictable effects on efficiency in the four blocks prior to the switch of cue-contingencies: We replicated the main effect of CONDITION in the univariate 4-level ANOVA (*F*(3,51) = 85.3, *p* < 0.001, *η*_*p*_^2^ = 0.83, 95% CI [0.78, 0.90]), with highest efficiency in easy trials (744.75 IES) and lowest efficiency in difficult trials (1212.46 IES); we also find a significant difference between medium conditions (higher efficiency in the low mean, low variance condition; 905.73 IES vs. 973.45 IES; *t*(17) = 2.4, *p* = 0.03, *r equivalent* = 0.38, 95% CI [0.15, 0.55]), as a result of faster, yet equally accurate responses in the low mean, low variance condition. To dissociate effects of (cue-driven) predicted confidence and stimulus characteristics on perceptual decision-making and performance confidence, we entered the average accuracy measured as percentage correct responses in Blocks 4 and 5 into a repeated-measures ANOVA with the factors CONDITION (4 levels) and SWITCH (2 levels: pre/switch; that is Block 4 vs. 5). We found no indication that participants performed better or worse in any condition after the switch of cue-condition contingencies. The repeated-measures ANOVA yielded the expected main effect of CONDITION (*F*(3,51) = 39.5, *p* < 0.001, *η*_*p*_^2^ = 0.70, 95% CI [0.63, 0.80]), but no main effect of SWITCH (*F* < 1), and no reliable interaction (*F*(3,51) = 1.1, *p* = 0.35, *η*_*p*_^2^ = 0.06, 95% CI [0.03, 0.25]). The corresponding repeated measures ANOVA on RTs yielded the same result, with the expected main effect of CONDITION (*F*(3,51) = 15.6, *p* < 0.001, *η*_*p*_^2^ = 0.48, 95% CI [0.27, 0.70]), but no main effect of SWITCH (*F* < 1), and no reliable interaction (*F*(3,51) = 1.8, *p* = 0.16, *η*_*p*_^2^ = 0.09, 95% CI [0.03, 0.28]).

##### Performance Confidence

Analysis of both performance confidence and predicted confidence was performed separately for blocks before and after the switch. Confidence measures prior to the switch, in blocks with the original cue-condition contingencies, constitute a replication of Experiment 1. The two blocks after the switch allow us to test two different aspects of our hypotheses: First, we hypothesized that changing the cue-condition contingencies will affect performance confidence, as previous associations led to confidence prediction errors. A direct comparison of confidence judgments in the last block before (Block 4) and the first block after the switch (Block 5), allows us to assess the influence of (old) confidence predictions on performance confidence.

In the first 4 blocks of the experiment, we replicated the differences in decision-confidence between conditions that were also included in Experiment 1: There was a significant effect of CONDITION in the 4-level univariate ANOVA (*F*(3,51) = 42.6, *p* < 0.001, *η*_*p*_^2^ = 0.71, 95% CI [0.64, 0.81]), and the paired-samples *t*-test between medium conditions likewise yielded a significant result (*t*(17) = 2.4, *p* = 0.03, *r equivalent* = 0.38, 95% CI [0.08, 0.64]), with lower performance confidence in the high mean, high variance condition than in the low mean, low variance condition (Fig. 2). See Supplementary Information an analysis of error awareness.

##### Predicted Confidence

Testing for the effect of CONDITION on predicted confidence, we found the predicted significant main effect in the 4-level univariate ANOVA (*F*(3,51) = 12.8, *p* < 0.001, *η*_*p*_^*2*^ = 0.43, 95% CI [0.30, 0.60]), showing that participants learned to predict how confident they typically felt following judgments on specific stimulus categories. The difference between medium conditions did not reach significance at the chosen alpha level of 5% (*t*(17) = 1.3, *p* = 0.20, *r equivalent* = 0.22, 95% CI [−0.09, 0.57]). The Bayes Factor in favor of this Null hypothesis of equality was *BF*_01_ = 1.90. To ensure that the non-significant effect on the difference between medium conditions (which was in the same direction as the significant effect in Experiment 1) did not point to substantial differences between the experiments, we pooled the data from both studies and conducted a 2 × 2 mixed-measures ANOVA with the between-subject factor EXPERIMENT (1/2) and the within-subject factor (MEDIUM CONDITION). This test showed that there was a significant effect of MEDIUM CONDITION on confidence across the entire pooled population (*F*(1,32) = 7.0, *p* = 0.01, *η*_*p*_^2^ = 0.18, 95% CI [0.04, 0.41]), but no significant effect of EXPERIMENT (*F*(1,32) = 2.6, *p* = 0.12, *η*_*p*_^2^ = 0.07, 95% CI [0.002, 0.26]) and, crucially, no reliable interaction (*F* < 1). Collectively, these results replicate the novel findings from Experiment 1 that people acquire category-specific confidence predictions.

#### Predicted Confidence Modulates Performance Confidence After the Cue-contingency Switch

**Figure 3:**
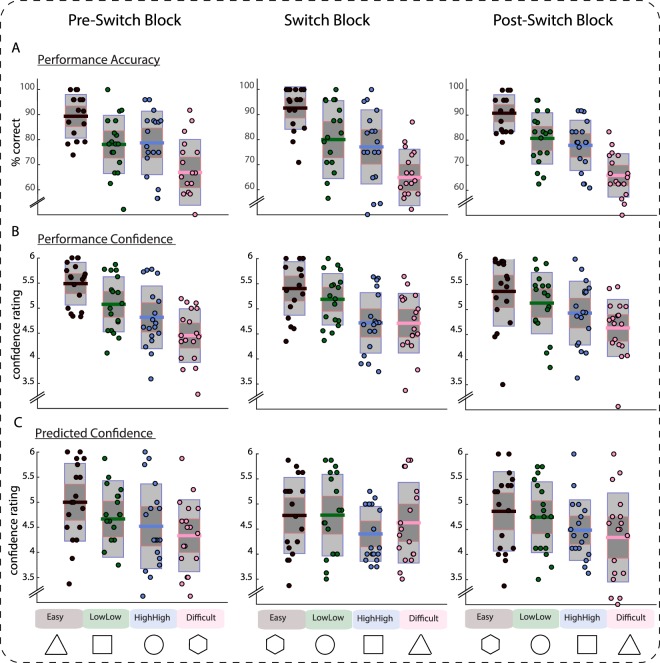
Accuracy (top), performance confidence (middle) and predicted confidence (bottom) in the last block prior to the switch (left), the switch block (middle column), and the block after the switch (right). Single dots show single-subject data, bars display mean of the distribution (solid line), 95% confidence interval (dark grey shaded area), and 1 standard deviation (light grey shaded area). Performance remains largely unaffected by the switch in cue-condition contingencies. Performance confidence for difficult trials is increased after the switch. Predicted confidence measures show adaptation to the new contingencies, with accurate predictions in the second block following the switch.

The results in Experiment 1, and in the initial blocks of Experiment 2, indicate that participants formed confidence predictions for the different condition cues. However, the impact of such cues on behavior in these blocks are impossible to disentangle from the behavioral effects of the conditions themselves, given the consistent association between cues and conditions. In contrast, in the post-switch adaptation blocks (Blocks 5 and 6), predictions and experience are set in conflict (at least until the new contingencies are learned). Thus, contrasting pre- and post-switch behavior (Fig. 3) enabled us to study the interaction between predicted confidence and performance confidence.

As shown in the lower panels of Fig. 3, adaptation of predicted confidence was a gradual learning process after the switch in cue-condition contingencies. Prior to the switch, predicted confidence closely tracked performance confidence and, but for the small but robust difference in confidence between medium conditions, also tracked objective accuracy. However, after the switch, predicted confidence varied much less systematically across conditions (Fig. 3, lower middle panel). These differences were apparent in a 4 × 2 repeated-measures ANOVA with the factors CONDITION (1–4) and SWITCH (pre/switch; that is Block 4 vs. 5), which yielded a significant effect of CONDITION (*F*(3,51) = 4.1, *p* = 0.01, *η*_*p*_^2^ = 0.20, 95% CI [0.07, 0.38]), no significant effect of SWITCH (*F* < 1), but a significant interaction (*F*(3,51) = 3.6, *p* = 0.02, *η*_*p*_^2^ = 0.18, 95% CI [0.08, 0.37]).

Thus, in the immediate post-switch block (Block 5), confidence predictions differed from experienced confidence across conditions. To investigate the effects of this interaction between prediction and experience, we analyzed participants’ performance confidence ratings in the blocks immediately before and after the switch (left and middle panel), using a 4 × 2 repeated-measures ANOVA with the factors CONDITION (1–4) and SWITCH (pre/switch; that is Block 4 vs. 5). This analysis revealed a significant main effect of CONDITION (*F*(3,51) = 22.0, *p* < 0.001, *η*_*p*_^2^ = 0.56, 95% CI [0.47, 0.68]), no significant effect of SWITCH (*F* < 1), but a significant interaction (*F*(3,51) = 6.7, *p* < 0.001, *η*_*p*_^2^ = 0.28, 95% CI [0.16, 0.48]).

As shown in Fig. 3 and Table 1, the variability of performance confidence across conditions was reduced following the switch of cue-contingencies, largely due to the fact that participants became more confident on difficult trials. Thus, pairwise contrasts between pre- and switch performance confidence for each condition separately revealed only a significant increase in confidence in difficult trials (which were preceded by cues by that previously preceded easy trials; *t*(17) = 3.5, *p* = 0.003, *r equivalent* = 0.51, 95% CI [0.21, 0.80]). No other differences were reliable (Table 1). This constitutes partial confirmation of our predictions, with learnt cue associations leading to increased confidence on difficult trials, but little evidence of a corresponding decrease in confidence on easy trials. We thus find that predicted confidence becomes integrated into performance confidence, although the effect does not reach significance in all conditions.

**Table 1:** Confidence ratings, compared by condition both between the last block prior to the switch and the switch block (left column) and the last block prior to the switch and the second block after the switch.

Pre-switch to Switch					Pre-switch to Post-switch				
Performance Confidence	*t*	*p*	*r*.*e*.	CI	Performance Confidence	*t*	*p*	*r*.*e*.	CI
Easy	−1.0	0.31	−0.18	−0.49, 0.15	Easy	−1.3	0.22	−0.21	−0.41, 0.11
Low, low	1.7	0.11	0.27	−0.01, 0.52	Low, low	0.50	0.62	0.09	−0.26, 0.44
High, high	−1.0	0.33	−0.17	−0.52, 0.16	High, high	1.6	0.14	0.26	−0.05, 0.56
Difficult	3.5	0.003	0.51	0.21, 0.80	Difficult	1.6	0.12	0.27	−0.03, 0.53
**Predicted Confidence**					**Predicted Confidence**				
Easy	−1.0	0.31	−0.18	−0.50, 0.15	Easy	−1.2	0.26	−0.20	−0.56, 0.14
Low, low	1.0	0.36	0.16	−0.17, 0.47	Low, low	0.56	0.58	0.10	−0.36, 0.34
High, high	−0.8	0.44	−0.14	−0.42, 0.21	High, high	−0.3	0.78	−0.05	−0.0, 0.28
Difficult	2.0	0.07	0.32	−0.01, 0.65	Difficult	0.04	0.97	0.01	−0.35, 0.34

Switch-effects (significant interaction) are carried by confidence changes in difficult trials. Positive *t*-values indicate average confidence values that are larger in the switch block than pre-switch block, or larger in the post-switch block than pre-switch block. All df = 17.

A final analysis concerned the hypothesized re-adjustment of confidence predictions, resulting from the mismatch between predicted and performance confidence in the switch block (Block 5). A visual description of these changes is shown in Fig. 3, in the rightmost panel. To this end we compare performance confidence between the last block before the switch (Block 4) and the last block following the switch (Block 6). The according repeated measures ANOVA revealed a main effect of CONDITION (*F*(3,51) = 22.1, *p* < 0.001, *η*_*p*_^2^ = 0.57, 95% CI [0.48, 0.69]), no main effect of SWITCH (*F* < 1), and contrary to the comparison with the switch block, the interaction between CONDITION and SWITCH did not reach significance at the chosen alpha level of 5% (*F*(3,51) = 2.3, *p* = 0.08, *η*_*p*_^2^ = 0.12, 95% CI [0.05, 0.28]). As evident in Fig. 3, numerically there was a decrease in confidence on difficult trials, reverting back towards the pre-switch levels. There were no significant differences between performance confidence judgments within a condition in the comparison of the pre-switch (Block 4) and the last post-switch block (Block 6; Table 1). As expected, we find an adjustment in predictions, too, showing that participants successfully re-learned predictions associated with each stimulus as a result of the experienced confidence. This re-learning of predictions is borne out by the repeated measures ANOVA, showing an effect of CONDITION (*F*(3,51) = 5.6, *p* = 0.002, *η*_*p*_^2^ = 0.25, 95% CI [0.13, 0.42]), but no main effect of SWITCH (*F* < 1), and no significant interaction (*F* < 1). Likewise, none of the within-condition, across-block comparisons reached significance (Table 1).

To conclude, we found a bi-directional link between predictive and performance confidence: Predictive confidence modulated performance confidence (evident in the switch block; Block 5), but was also adapted as a result of mismatches between predicted and experienced confidence (evident in the final block; Block 6).

#### Summary of Behavioral Results

The central finding of Experiment 1 - that participants can acquire cue-based confidence predictions - was replicated here. Confidence predictions closely tracked reported performance confidence, even when performance confidence dissociated from objective accuracy. Experiment 2 further showed that learned confidence predictions can modulate performance confidence, despite a lack of reliable change in objective performance. Finally, confidence predictions were gradually updated following confidence prediction errors in feedback-free environments, and concurred with performance confidence in the second block after the switch in cue contingencies (Block 6).

#### EEG

The critical measures for the EEG analysis were the amplitudes of the CNV, a pre-stimulus slow-wave component that reflects preparation for stimulus processing, and the CPP, a post-stimulus centro-parietal component that reflects external and internal effects on decision making in its slope and amplitude. Analysis of the EEG data follows the behavioral analysis, focusing on the effect of CONDITION in a univariate ANOVA and differences between medium conditions in paired *t*-tests to assess the effects of confidence on neural signatures of stimulus preparation and processing.

##### CNV

The first question addressed with the EEG analysis was whether confidence predictions would modulate preparation for stimulus processing. The CNV is a useful marker of such covert internal preparation. CNV amplitude was quantified in 4 adjacent time-windows, each 100 ms, covering the 400 ms prior to stimulus onset. ERPs were measured across an electrode cluster consisting of C3 – CZ – C4 – CP3 – CPZ – CP4 – P3 – PZ – P4 in the first 4 blocks of the experiment (in the *acquisition* phase, before cue-condition contingencies switched). We expected CNV amplitude to scale with predicted confidence, as larger amplitudes are associated with a greater readiness to process and respond to a stimulus^20,27^. We therefore predicted the highest amplitude following cues associated with easy trials and lowest amplitude following cues associated with difficult trials. We also tested for a difference in amplitude between cues indicating the medium conditions, in line with the differences between predicted confidence.

**Figure 4:**
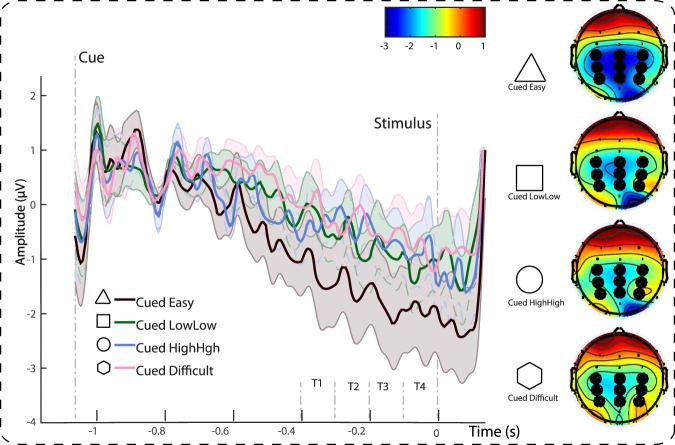
Time-voltage plots and scalp topographies showing the differences in CNV amplitude across conditions. Statistical analyses rely on the 4 time windows (T1-T4) prior to stimulus onset. Data are not smoothed for display purposes. Shaded error bars depict the standard error of the mean. Scalp topographies show the average amplitude across T4 (100 to 0 ms prior to response), thresholded between −3 and 1 µV.

The first-pass analysis therefore included one 4-level factor for the 4 cue CONDITIONs, one 4-level factor for the 4 TIMEWINDOWs, and one 3-level factor for the 3 degrees of POSTERIORITY (1: C3 – CZ – C4, 2: CP3 – CPZ – CP4, 3: P3 – PZ – P4) to assess the scalp topography of observed effects. This repeated-measures ANOVA yielded the expected main effect of TIMEWINDOW (*F*(3,51) = 8.8, *p* < 0.001, *η*_*p*_^*2*^ = 0.34, 95% CI [0.11, 0.48]) with an increase in amplitude over time, a significant main effect of CONDITION (*F*(3,51) = 3.0, *p* = 0.04, *η*_*p*_^*2*^ = 0.15, 95% CI [0.00, 0.29]), with an increase in amplitude from difficult to difficult easy trials, and no reliable effect of POSTERIORITY (*F* < 1). There was a significant interaction between TIMEWINDOW and POSTERIORITY (*F*(6,102) = 6.8, *p* < 0.001, *η*_*p*_^*2*^ = 0.28, 95% CI [0.11, 0.38]), as the negative amplitude was larger at more posterior electrodes, but no other reliable interactions (*F*s < 1). The main effect of condition was driven by an increase in CNV amplitude following easy cues (Fig. 4). Easy cues elicited the largest (negative) amplitude; paired *t*-tests showed that the amplitude difference between easy and difficult trials was statistically significant (*t*(17) = 2.4, *p* = 0.03, *r equivalent* = 0.39, 95% CI [0.10, 0.65]). Given this association with predictive confidence, we might expect greater CNV amplitude following cues associated with low mean, low variance trials than following cues associated with high mean, high variance trials. However, although a small numerical trend in the expected direction was observed (−1.14 μV vs. −0.98 μV), the difference was not significant (*t* < 1). The Bayes Factor in favor of this Null hypothesis of equality was *BF*_01_ = 3.54.

##### CPP

The second neural marker of interest, the centro-parietal positivity (CPP), was used to assess whether the neural correlates of stimulus processing would be modulated by confidence and performance. Focusing on the electrode cluster CZ – CPZ – PZ, we measured CPP amplitude as participants’ individual peak between 180 to 80 ms before the response (calculated separate for each electrode), and slope as a participant-wise linear fit to the data between 400 to 100 ms before the response (averages across electrodes; cf.^21,22^). To estimate effects of RT on the component, all trials were sorted by each participant’s median RT in each specific condition, creating two equal sized cells of fast versus slow trials in each condition.

The CPP slope is associated with internal and external influences on the sampling process that also affect RTs, while amplitude is suggested to reflect an internally regulated decision threshold^21,22^. We thus expected RTs to account for the larger part of the variance in the slope of the CPP, with a smaller effect of stimulus condition^22^. Further, we expected to see differences in CPP amplitude across conditions above and beyond differences driven by RT, in line with the result of the drift-diffusion model of the data, which suggests that decision thresholds vary between conditions (see Supplementary Information). Finally, we tested whether these differences between conditions would be affected by expectations, exploiting the dissociation between expectations and actual conditions provided by the switch in cue-condition contingencies.

**Figure 5:**
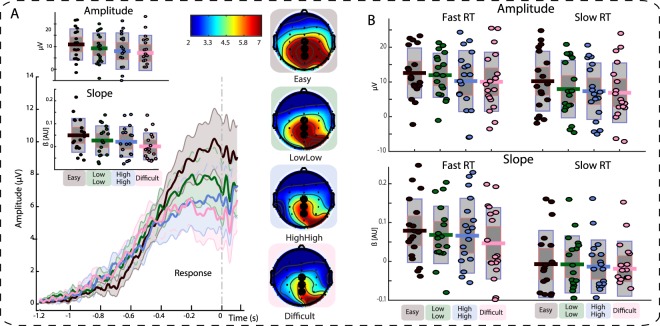
(**A**) Time-voltage plots and scalp-topographies showing the CPP in the blocks prior to the switch in cue-condition contingencies. Inserts show single-subject data for CPP amplitude and slope across conditions. Scalp topographies show voltage averages 180 to 80 ms prior to response. All topographies are thresholded between 2 and 7 µV. Data are not smoothed for display purposes. Shaded error bars depict the standard error of the mean. (**B**) CPP amplitude and slope plotted on the single-subject level for fast and slow trials separately. Single dots show single-subject data, bars display mean of the distribution (solid line), 95% confidence interval (dark grey shaded area), and 1 standard deviation (light grey shaded area). Amplitude differences across conditions show little variation by RT, however, RT does modulate differences in slope across conditions. Time-window for Amplitude: 180 to 80 ms prior to response. Time-window for calculation of Slope: 400 to 100 ms prior to response.

Slope showed a significant effect of CONDITION (Fig. 5a) in the univariate ANOVA (*F*(3,51) = 6.2, *p* = 0.001, *η*_*p*_^2^ = 0.27, 95% CI [0.14, 0.48]). However, when trials were split by fast and slow RTs, we found that, as expected, most of that effect can be attributed to the difference in stimulus processing: A repeated-measures ANOVA with the factors RT (fast vs. slow) and CONDITION showed a significant effect of RT (*F*(1,17) = 15.8, *p* < 0.001, *η*_*p*_^2^ = 0.48, 95% CI [0.28, 0.69]), whereas the effect of CONDITION did not reach significance at the chosen alpha level of 5% (*F*(3,51) = 2.3, *p* = 0.09, *η*_*p*_^2^ = 0.12, 95% CI [0.04, 0.33]). There was furthermore no reliable interaction (*F*(3,51) = 0.49, *p* = 0.69, *η*_*p*_^2^ = 0.03, 95% CI [0.01, 0.21]; Fig. 5b).

Analysis of the trials with neutral cues, which do not predict stimulus category, yielded no statistically significant difference in slope between conditions. The univariate ANOVA testing the 4-level factor CONDITION revealed no reliable effect of stimulus category (*F*(3,51) = 2.0, *p* = 0.13, *η*_*p*_^2^ = 0.11, 95% CI [0.03, 0.31]). Because this analysis involves fitting linear trends to a small set of data points (only 20% of trials were preceded by neutral cues), this null-effect is inconclusive as it may have been caused by a disproportionately small sample size (low trial numbers) and therefore a poor signal-to-noise ratio, compared to other analyses.

**Figure 6:**
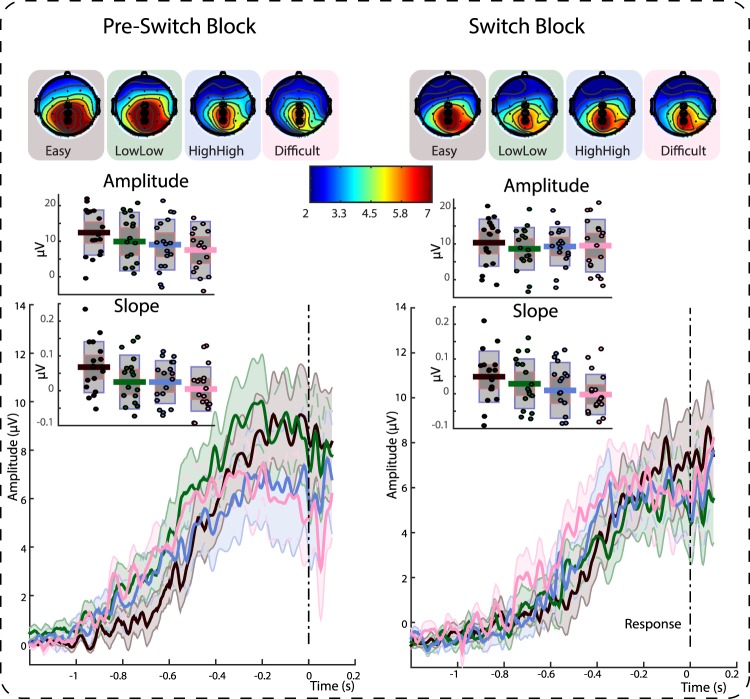
Time-voltage plots and scalp-topographies showing the CPP in the last block prior to the switch in cue-condition contingencies (left), and in the switch block (right). Inserts show single-subject data for CPP amplitude and slope across conditions. Single dots show single-subject data, bars display mean of the distribution (solid line), 95% confidence interval (dark grey shaded area), and 1 standard deviation (light grey shaded area). Both measures reflect conditions, and amplitude shows more modulation by the switch. Scalp topographies show voltage averages across 40 ms prior to response. All topographies are thresholded between 2 and 7 µV. Data are not smoothed for display purposes. Shaded error bars depict the standard error of the mean. Time-window for Amplitude: 180 to 80 ms prior to response. Time-window for calculation of Slope: 400 to 100 ms prior to response.

We were interested in amplitude differences driven by expectations around the switch in cue-condition contingencies, and for the sake of completeness also performed the same repeated measures ANOVA on the slope data focusing on the last block prior to the switch (Block 4) and the switch block (Block 5). We found a significant effect of CONDITION (*F*(3,51) = 7.8, *p* < 0.001, *η*_*p*_^2^ = 0.31, 95% CI [0.13, 0.54]) but only a numerical trend of cue-contingency SWITCH that did not reach significance at the chosen alpha level of 5% (*F*(1,17) = 3.2, *p* = 0.09, *η*_*p*_^2^ = 0.16, 95% CI [0.01, 0.46]) and no reliable interaction (*F* < 1). Note that this analysis does not control for the effects of RT (Fig. 6).

Together these results replicate the finding that CPP slope is modulated by the internal and external influences on perceptual decision making that affect RTs. It remains unclear, however, which, if any, modulation arises from expectations of the specific stimulus condition.

A drift-diffusion model analysis of the present two datasets (see Supplementary Information; see also^28^) suggests that participants adjusted their decision threshold for each condition. We tested whether this behavior was also reflected in the CPP amplitude, a neural marker of evidence-to-bound accumulation. In a first step, a repeated measures ANOVA with factors of CONDITION (4 levels) and POSTERIORITY (3 levels; 1: CZ, 2: CPZ, 3: PZ) revealed a main effect of CONDITION (*F*(3,51) = 5.2, *p* = 0.03, *η*_*p*_^2^ = 0.23, 95% CI [0.10, 0.46]) and a main effect of POSTERIORITY (*F*(2,34) = 4.4, *p* = 0.02, *η*_*p*_^2^ = 0.21, 95% CI [0.04, 0.56]; Fig. 6), but no reliable interaction (*F* < 1). Numerically, easy trials lead to the largest CPP amplitude, followed by low mean, low variance trials, high mean, high variance trials, and finally difficult trials with the lowest CPP amplitude (Fig. 5a).

Pairwise comparisons between neighboring conditions showed that the only significant difference in CPP amplitude was between easy and low mean, low variance trials (*t*(17) = 2.4, *p* = 0.03, *r equivalent* = 0.38, 95% CI [0.08, 0.63]). In particular, there was no difference between medium conditions (*t*(17) = 1.3, *p* = 0.22, *r equivalent* = 0.22, 95% CI [−0.11, 0.62]). The Bayes Factor in favor of this Null hypothesis of equality was *BF*_01_ = 2.03. This pattern corresponds to modeled decision boundaries, which were also amplified for easy trials (see Supplementary Information).

In contrast to the findings for CPP slope, the amplitude differences were not sufficiently explained by differences in RT. Trials sorted into slow and fast trials by median split were entered into a 4 × 2 repeated measures ANOVA, yielding a significant main effect of CONDITION (*F*(3,51) = 6.9, *p* < 0.001, *η*_*p*_^2^ = 0.29, 95% CI [0.14, 0.53], and a main effect of RT (*F*(1,17) = 6.7, *p* = 0.02, *η*_*p*_^2^ = 0.28, 95% CI [0.07, 0.57]), with no interaction (*F* < 1; Fig. 5b).

Switching cue-condition contingencies in the later stages of our experiment provided us with a test for the effect of predicted confidence on neural correlates of stimulus processing. A repeated measures ANOVA with the factors CONDITION (4 levels) and POSTERIORITY (3 levels; 1: CZ, 2: CPZ, 3: PZ) and SWITCH (pre/switch; that is Blocks 4 and 5) revealed a main effect of CONDITION (*F*(3,51) = 3.8, *p* = 0.01, *η*_*p*_^2^ = 0.18, 95% CI [0.01, 0.33]) and a main effect of POSTERIORITY (*F*(2,34) = 4.8, *p* = 0.02, *η*_*p*_^2^ = 0.22, 95% CI [0.01, 0.41, and no main effect of SWITCH (*F* < 1; Fig. 6). Moreover, contrary to our hypothesis, there was no significant interaction between CONDITION and SWITCH (*F*(3,51) = 2.1, *p* = 0.11, *η*_*p*_^2^ = 0.11, 95% CI [0.00, 0.24]). None of the other interactions were significant (*F*s < 1.8, *p*s > 0.11, *η*_*p*_^2^s < 0.09). Because behavioral confidence effects emerge primarily on difficult trials comparing pre- and switch confidence, and in the interaction between SWITCH and CONDITION when only the easy and difficult conditions are included, we conducted an exploratory analysis targeting these conditions. The repeated measures ANOVA included the factors POSTERIORITY (3 levels; 1: CZ, 2: CPZ, 3: PZ) and SWITCH (pre/switch; that is Blocks 4 and 5), and a 2-level factor CONDITION (easy/difficult). This analysis yielded a main effect of CONDITION (*F*(1,17) = 6.9, *p* = 0.02, *η*_*p*_^2^ = 0.29, 95% CI [0.01, 0.54]), a main effect of POSTERIORITY (*F*(2,34) = 3.8, *p* = 0.03, *η*_*p*_^2^ = 0.18, 95% CI [0.00, 0.37) and as expected no main effect of SWITCH (*F* < 1), but the expected interaction between SWITCH and CONDITION was significant (*F*(1,17) = 6.0, *p* = 0.03, *η*_*p*_^2^ = 0.26, 95% CI [0.00, 0.52]). No other comparisons reached significance (*F*s < 2.0, *p*s > 0.15, *η*_*p*_^2^s < 0.11). The interaction between SWITCH and CONDITION is explained by the same numerical changes as the interaction between SWITCH and CONDITION in the confidence data (cmp. Figs 3 and 6): Difficult trials show a numerically larger CPP amplitude in the switch block compared to the pre-switch block which however did not reach significance at the chosen alpha level of 5% (7.52 µV vs. 9.49 µV; *t*(17) = 1.9, *p* = 0.07, *r equivalent* = 0.32, 95% CI [0.02, 0.56], *BF*_01_ = 0.89), whereas easy trials show a numerically smaller CPP amplitude in the switch block compared to the pre-switch block, also not reaching significance (12.42 µV vs. 10.34 µV; *t*(17) = 1.8, *p* = 0.09, *r equivalent* = 0.29, 95% CI [−0.04, 0.64], *BF*_01_ = 1.11).

Finally, analysis of trials in which cues were non-predictive of the stimulus-category also yielded the same pattern of differing amplitude levels across conditions as all other comparisons. The univariate ANOVA testing the 4-level factor CONDITION showed the expected main effect (*F*(3,51) = 11.8, *p* < 0.001, *η*_*p*_^2^ = 0.41, 95% CI [0.33, 0.57]). Pairwise comparisons of neighboring conditions show a significant differences only between difficult and high mean, high variance stimuli (*t*(17) = 3.3, *p* = 0.004, *r equivalent* = 0.49, 95% CI [0.26, 0.71]), but no other significant results in paired comparisons between neighboring conditions (easy – low low: *t* < 1, *BF*_01_ = 2.84; low low – high high: *t*(17) = 1.9, *p* = 0.08, *r equivalent* = 0.30, 95% CI [−0.01, 0.53], *BF*_01_ = 1.01). We thus find differences in stimulus processing driven by the stimulus condition, regardless of whether the condition was expected on a given trial.

#### EEG Summary

To summarize, we established that predicted confidence drives neural preparation for stimulus processing as reflected in the CNV. Stimulus condition showed a modulating effect on the CPP, a neural maker of decision-making, in line with differences in performance. While small differences in performance confidence, such as associated with the medium conditions, led to differences in predicted confidence, we could not conclusively establish an effect of these subtle differences in confidence on neural *preparation* of stimulus processing in the CNV. Please refer to the Supplementary Information for an alternative analysis of the EEG data, which yields comparable results.

## Discussion

In the present study, we addressed the question of whether people learn about their own confidence in perceptual decisions, evident in confidence predictions. More specifically, we hypothesized that predicted confidence would closely follow actual performance confidence, rather than objective accuracy. Our findings support this hypothesis: People quickly learned to associate predicted confidence with visual cues in a perceptual categorization task, showing the same systematic biases as in their performance confidence. As expected, predicted confidence in turn affected performance confidence, as evident after sudden, un-announced changes in cue-stimulus contingencies. This finding points towards the relevance of expectations as additional sources of information (cues) that become integrated in confidence estimates. This expands our understanding of the multi-cue nature of this metacognitive evaluation, in showing that information beyond physical features of the stimulus and subsequent properties of the decision process are taken into account. Finally, the findings from our EEG analyses suggest that predicted confidence affects neural preparation for and neural processing of the visual task, showing that confidence predictions affect task preparation. Our study has thus strong theoretical implications for the role of metacognitive judgements in task preparation and performance evaluation.

In recent years, a large body of empirical evidence has accrued suggesting that people are capable of accurately tracking their own performance, even in the absence of feedback (e.g.^29,30^). Despite these advances, dominant theories of metacognition still struggle to explain how, and based on what information, confidence signals are formed (for recent accounts see^1,2,7,31^). In the present study, we extended previous accounts of metacognition in two important ways: First, our findings suggest that performance confidence is at least to some extent based on predicted confidence (see also^13^). Second, we propose that confidence predictions can be used to prepare for the uncertainty of the upcoming decision.

Regarding the first implication of our work: Performance confidence across conditions becomes more similar following the switch in cue-condition contingencies when predictions and actual conditions do not match up - this shows that expectation influences experience in the domain of confidence. In detail, participants were overly confident regarding their performance on difficult trials, when these were initiated by cues that were associated with an easy stimulus. This finding suggests that predicted confidence - in addition to other informative cues such as experienced difficulty - feeds into performance confidence. This interpretation fits a hypothesis developed in the context of metacognition in memory (*metamemory*), which proposes that metacognitive judgements are based on multiple heuristic cues^32,33^ such as the familiarity of the question^34^ and the accessibility of information at retrieval^35^. We have recently proposed a similar mechanism for decision confidence, suggesting that confidence is not just purely driven by the evidence that forms the basis of the decision but also other heuristic cues, such as the reliability of evidence or decision speed^7^. Here, we provide important evidence that one of the multiple cues informing confidence judgments are experience-based expectations regarding task performance.

The results neatly fit a Bayesian account of human cognition, where experience leads to the formation of priors. Once prior beliefs exist, they inform all inferences about the true state of the world, i.e. the posterior belief. We can maintain accurate inferences about the environments we act in, because posterior beliefs also integrate observable evidence, and in turn affect future priors. Translated to the current study, we show that confidence itself, after repeated experience, becomes a prior belief on performance in the task. The decision itself then provides information that together with the prior is incorporated in a posterior belief in task-performance. It remains for future work to develop mathematical formalizations of this framework. Such formalizations would need to address the key question of whether common or distinct representations underpin the different confidence readouts used in the present study, and the related question of whether these readouts are informed by at least partially distinct cues (cf. metamemory research where prospective feeling of knowing judgments and retrospective confidence judgments are thought to be at least partially dissociable in terms of their computational basis and adaptive uses, even if both reflect the same underlying construct of the availability of information in memory^36,37^.

An implication of findings in line with this theoretic account is the idea that the utility of confidence is not limited to evaluating past decisions. Decision confidence is often regarded as an internal proxy for feedback^38–40^. Here, we argue that in addition to such self-evaluative, post-hoc weighting of evidence based on how uncertain we feel about a recent decision, confidence could also be useful in anticipating uncertainty or effort of an upcoming decision. Such prospective metacognitive judgements have been understudied in the context of decision making (see also^41^; but see^13^). In contrast, research on metamemory has focused extensively on predictive judgments. This research has established, for instance, that *ease-of-learning judgements* (EOLs;^10^) and *judgements of learning* (JOLs;^12^) regulate the allocation of study time. Here, we build upon this work, and show an important conceptual transfer: Predicted confidence appears to also play a role in the context of perceptual decision making.

Taken together, our results provide an important step towards determining how metacognitive judgements are formed internally, and may be utilized in the future, thus extending the classic frameworks by including predicted confidence. The multi-cue model of confidence that we propose here links the field of decision confidence with a line of research focusing on metamemory, highlighting that similar mechanisms might generate metacognitive signals in the case of both choices and memory. Future studies should aim to shed light on which other cues feed into confidence signals and also how precisely predicted confidence is formed.

Moreover, the idea that expectation and experience of confidence inform each other continuously makes sense if we assume that predicted confidence in particular guides how much effort people invest into individual choices (see also^11,42^). The notion that confidence predictions can be used to guide behavior of course also applies to other examples of metacognitive control, for instance allocation of study time^10,12^, and decisions about whether to rely on one’s prospective memory or to set external reminders to complete future tasks^43^.

The relevance of predictions for perceptual decision making has been highlighted in various contexts. On the one hand, expectations of perceptual events themselves influence preparation for and processing of these stimuli (^44^ for a review). On the other hand, it has been shown that characteristics associated with these perceptual events such as their reward value affect behavior, including oculomotor responses in visual search^45,46^. Our study extends this view substantially by showing that, in the wider context of learning, not only external events but also internal evaluation of performance affects expectations. Internal models require continuous updating to remain valid and useful vehicles of preparation^47^.

The present study shows that prediction and experience inform each other, and that this continuous update allows accurate assessments of the environment. These findings furthermore match the results of a recent study by Fleming and colleagues^13^, who found that both predicted confidence as well as performance confidence were at least to some extent informed by the other type of judgment on the previous trial. This effect was stronger for predicted confidence, which was largely driven by the experienced confidence on the previous trial. However, it should be noted that the task used in this study kept difficulty constant using a staircase procedure and that participants had no context-specific cues that could help them anticipate the upcoming decision.

A related idea has recently been investigated in a study by Guggenmos and colleagues^40^. The authors investigated the role of confidence in reinforcement learning, suggesting that presently experienced confidence is judged constantly against expected confidence, thus serving as a proxy for external feedback. One important way in which our study extends this work is that, in contrast to this previous work^40^, we measured predicted confidence prior to the onset of the visual target of perceptual-decision making, thus measuring a truly experience-based, predictive signal.

Our findings furthermore suggest that predicted confidence leads to changes in stimulus processing, reflected in two key EEG correlates. Our first analysis targeted the CNV - a measure of preparatory activity. CNV amplitude increased in anticipation of a highly-confident trial. Second, we focused on the CPP - a neural correlate of perceptual processing thought to reflect evidence accumulation to a pre-set threshold^21^. In addition to replicating several key findings reported by O’Connell and colleagues^21^ (see also^22^), we found the CPP to vary with stimulus condition. Additional analyses ruled out that this CPP effect was driven by differences in reaction time and thus trial-by-trial variations in perceived difficulty. This supports the interpretation that this finding reflects modulation of neural processing through expectations. This interpretation is furthermore supported by the finding that the clear pattern of stimulus condition was diminished immediately after the switch in cue-condition contingencies, similar to the changes in confidence after the switch.

One of the key findings of the original study by O’Connell and colleagues^21^ was that CPP waveforms are characterized by a fixed amplitude, similar to a pre-set decision threshold. Our finding of a variation in CPP amplitude by condition stands in contrast to these results and instead suggests that participants adjusted their decision threshold flexibly across stimulus conditions. It should be noted that the propensity to resort to such a strategy could have been increased in our specific paradigm by presenting people with valid cues, allowing them to more flexibly prepare stimulus processing. A condition-contingent variation in decision boundary was similarly found in a separate diffusion model analysis, further supporting this interpretation of our data.

There is an apparent contradiction in our EEG findings reported in this study: While our CNV results could suggest that people were more ready to process the stimulus when they expected the upcoming decision to be easy compared to difficult, our CPP results (and the additional drift-diffusion model findings) seem to suggest the opposite, with a higher decision threshold for easy compared to difficult stimuli and thus a more cautious response mode. There are two possible explanations for this seemingly contradictory pattern of results. First, it is important to point out that the CNV is not a motor readiness potential (cf. the Bereitschaftspotential) - its amplitude does not reflect primarily the motor preparation to make a quick response, but is more likely associated with preparation of cognitive aspects of the upcoming task^20^. In fact, one possibility is that just as the stimulus-preceding negativity, a correlate found in anticipation of feedback, the CNV scales with expected information in the stimulus^48,49^. Another, even more interesting alternative is that the divergent pattern could be interpreted as evidence for the possibility that participants dynamically adjust the duration spent on different stages of the decision making process. More specifically, the speeding of sensory processes for trials expected to be easy (CNV finding), might have automatically have ‘freed up’ additional time for more cautious sampling behavior (CPP finding). Therefore the two findings are not necessarily contradictory. Moreover, such an increase in sampling behavior was not visible in overall RT differences between conditions, thus future studies should investigate this possibility using careful mental-chronometry techniques to disentangle the effects of expectation on different stages of the decision-making process. Taken together, we found that predicted confidence had an effect on neural correlates of stimulus preparation and neural correlates of stimulus processing, highlighting the relevance of this internal estimate for the neural computations underlying perceptual decision making.

The present study sheds new light on perceptual decision-making in the absence of feedback, highlighting the active role confidence seems to play in decision-making processes. In this study, we focused on a novel metacognitive measure, predicted decision confidence. Our results suggest that confidence integrates information from various sources, and affects neural processing profoundly.

## Methods

All methods were carried out in accordance with the relevant guidelines and regulations pertaining to non-clinical human research, as instructed by the institutional review board. Methods and procedures were approved by the Medical Sciences Inter-Divisional Research Ethics Committee, Medical Sciences Divisional Office, Oxford. All data was collected at the Department of Experimental Psychology in Oxford. All participants provided written informed consent.

### Participants

We tested 16 participants in the behavioral experiment (Experiment 1) and 20 participants in the EEG experiment (Experiment 2). Participants took part in only one of the experiments. Two participants were post-hoc excluded from Experiment 2, one because of excessive noise in electrodes crucial to the analysis, the other because the staircasing procedure failed and the participant displayed behavior substantively different from any other of the 35 participants (see Supplementary Information for a separate analysis of this dataset).

In both experiments, participants sat in an electrically shielded, sound attenuating booth to minimize distraction and artifacts in the EEG recordings. Stimuli were presented on a 20″ CRT monitor with a 75 Hz refresh rate using the MATLAB toolbox Psychtoolbox 3 with a 70 cm viewing distance^50–52^. All responses were made with a USB keyboard. The color judgments were made with the “c” or “n” key (left or right thumb). Confidence responses were made with the upper number line (keys “1”, “2”, “3”, “8”, “9”, and “0”) using the index, middle and ring fingers of the two hands.

### Statistical analysis

All statistical analysis was conducted in Matlab using the Measures of Effect Size (MES) Toolbox^53^. Where possible, we report point-biserial correlation coefficients (*r equivalent*). In addition to standard RT and accuracy measures, we compare performance across conditions as inverse efficiency, calculated as median correct RT divided by accuracy (inverse efficiency score IES^26^). Moreover, our design included two conditions of medium difficulty, which our previous studies had found to be matched in performance^7,19^. We therefore report Bayes Factors (*BFs*) for all non-significant *t*-tests to be able to assess the probability with which the null hypothesis is true given the data, using the *R* package BayesFactor by Morey, Rouder and colleagues^54,55^. We report the *BF* as support for the null hypothesis over the alternative hypothesis as *BF*_01_. Figures are based on a variation of the notBoxPlot function^56^.

### Staircase procedure

Participants completed extensive training in the perceptual decision task, both with and without confidence judgements (400 trials), during which an adaptive procedure was used to match the medium conditions. Predictive cues were introduced after 300 trials, to make sure that participants were able to perform the basic task well before having to deal with this increased complexity. In detail, conditions were matched with regard to percent accuracy. At the beginning of the staircase blocks (practice blocks two to eight), mean evidence in the low mean evidence, low variance condition was adjusted if accuracy between the two medium conditions differed in the preceding block. During this staircase procedure, participants were told that they were currently completing practice blocks, but were not told about the performance-adjusted increase or decrease in difficulty.

### EEG recording and pre-processing

A Neuroscan Synamps2 system (10 GΩ input impedance; 29.8 nV resolution; Neuroscan, El Paso, TX, USA)^57^ was used to record EEG data from 32 Ag/AgCl electrodes mounted in an elastic cap at locations FP1, FPZ, FP2, F7, F3, FZ, F4, F8, FT7, FC3, FCZ, FC4, FT8, T7, C3, CZ, C4, T8, TP7, CP3, CPZ, CP4, TP8, P7, P3, PZ, P4, P8, POZ, O1, OZ, and O2. Additional six external electrodes were attached: to the outer canthi of the left and right eyes, above and below the right eye to measure electro-oculograms (EOGs), and to the left and right mastoids. Electrode recordings were referenced to the right mastoid. All electrode impedances were kept below 50 kΩ. EEG data were recorded at a sampling rate of 1000 Hz. Data were online high-pass filtered at 0.1 Hz to avoid slow-wave drifts. The data were low-pass filtered at 24 Hz with a Hamming-windowed sinc finite impulse response function, as implemented in EEGLab^58–60^ prior to epoch extraction to avoid noise in the recording to inflate peak-measures of the components of interest. All analyses were performed on data down-sampled to 250 Hz. Auto-detected noisy channels were removed, and interpolated using the EEGLab pop_rejchan function^58^. We extracted cue-epoched data from 300 ms pre cue to 2800 ms post cue. These epochs were baselined to a window 100 to 200 ms post-cue, to prevent differences in the visual response to the stimuli affecting the baseline. Epochs with noise in the component-specific target electrodes were identified using first a probabilistic (pop_jointprob) followed by a thresholding (pop_eegthresh) approach (both implemented in EEGLab^58^). This excluded epochs that either fell outside a 95% confidence interval or had voltage differences ranging above or below 50 µV from the baseline (i.e. changes of more than 100 µV per epoch).

### EEG analysis

The EEG analysis focused on two neural markers of perceptual decision-making, which are associated with stimulus preparation and stimulus processing, respectively. The first target component was a preparatory negative-going potential in anticipation of the stimulus, following the cue: the contingent-negative variation (CNV). The CNV is a slow-wave component, associated with the readiness to respond^27^.

We measured the preparatory potential as the average voltage amplitude in four time-windows preceding the stimulus: −399 ms to −300 ms, −299 ms to −200 ms, −199 ms to −100 ms, and −99 ms to stimulus onset. The preparatory potential was estimated across an electrode cluster containing the electrodes C3 - CZ - C4 - CP3 - CPZ - CP4 - P3 - Pz - P4. We chose this large cluster as the amplitude of the CNV and related preparatory potentials typically varies across parietal and frontal electrodes^20,61–63^; we included posteriority of the electrode within the cluster as a factor in our analyses to assess scalp topography of observed effects. We predicted that CNV amplitude would scale with the levels of predicted confidence, hypothesizing that the expectation of a high confidence would lead to a greater readiness to respond to the stimulus. We therefore expected cues indicating easy trials leading to the largest CNV (most negative amplitude), and cues indicating difficult trials leading to the smallest CNV (least negative amplitude).

The second component we focused on was the centro-parietal positivity at the time of the response to the perceptual decision (CPP)^21,22^. The CPP is associated with the sampling of available evidence in perceptual decisions and peaks at the time of the response. Our CPP analysis focused on two main features of the component, taken to reflect different aspects of the decision-making process: The slope of the CPP is suggested to reflect sampling of the perceptual evidence, whereas its amplitude is taken to reflect the internal threshold for accumulated evidence that needs to be reached for a decision to be made^21,22^.

Our first aim was to replicate the finding that a steeper slope of the component accompanies faster reaction times. We further tested whether the condition in which a stimulus appeared would affect the slope of the component, above and beyond the variance explained by RTs^22^. Similarly, we hypothesized that CPP amplitude would vary between conditions, independent of RTs, as suggested by differential decision thresholds between conditions; these differential thresholds were indicated by a drift-diffusion model analyses we performed on the present data and in past modeling work within the paradigm^28^; [Supplementary Information]. Second, our design allowed us to test the idea that stimulus processing can be affected not only by the stimulus features (condition), but also by expectations about conditions. We used this setup to test for expectation-driven modulations of CPP amplitude by comparing identical conditions pre- and post-switch; we expected an interaction between the effects of condition and switch on CPP amplitude.

A post-cue baseline avoided contamination of the measurement by cue-driven variation (see below). Using trial-by-trial reaction time recordings the data were realigned to the individual responses. We measured CPP slope by fitting a linear regression to each participant’s individual data in a window ranging from 400 ms to 100 ms before the response. CPP amplitude was measured as the peak in a time-window ranging from 180 ms to 80 ms before the response^21^ in an electrode cluster containing the electrodes CZ-CPZ-PZ^21^.

For the CPP analysis of pre-switch blocks, the lowest number of trials entered per participant was 44, the average was 93. For the CNV analysis, the lowest number of trials per participant per condition was 41, the average was 72. Because the CNV was measures across a larger time-window, epochs were more likely to contain noise and therefore be discarded (see Supplementary Table 3 for details). Please refer to the Supplementary Information for an analysis that included data that was pre-processed identically, with the addition of an extra step, a current source density correction (*Current Source Density Corrected*).

### Code availability statement

All analysis code is available from the lead author on request.

## Supplementary information

Supplementary Information

## Data Availability

Data are available from the lead author on request.
