# Supplementary material for: Confidence Predictions Affect Performance Confidence and Neural Preparation in Perceptual Decision Making
Source: Sci Rep. 2019 Mar 11;9:4031. doi: 10.1038/s41598-019-40681-9 (PMC6411854; doi:10.1038/s41598-019-40681-9)
Supplement: Supplementary file 1 — Supplementary Information [file 41598_2019_40681_MOESM1_ESM.pdf]

Confidence Predictions Affect Performance Confidence and Neural Preparation in Perceptual Decision Making.

Annika Boldt [1,2,3,*], Anne-Marike Schiffer[1,4,*], Florian Waszak[5,6] and Nick Yeung[1]

[1]Department of Experimental Psychology, University of Oxford, OX1 3UD, UK, [2]Department of Psychology, University of Cambridge, CB2 3EB, UK, [3]Institute of Cognitive Neuroscience, University College London, UK,[4]Division of Psychology, Department of Life Sciences, Brunel University London, Uxbridge, UK, [5]Université Paris Descartes, Sorbonne Paris Cité, Paris, France [6]Laboratoire Psychologie de la Perception, UMR 8158, Paris, France

Supplementary Information

Supplementary Table 1: Pairwise comparisons across stimulus conditions for key behavioral measures in Experiments 1 and 2; please note that for consistency all comparisons are right-sided t-tests. This means that differences in line with the hypotheses have negative t-values and effect sizes in the RT and efficiency measure, where smaller numbers correspond to better performance.

	**Experiment 1** (df=15)					**Experiment 2** (df =17)				
Accuracy	t	p	r.e.	CI		t	p	r.e.	CI	
Easy vs LowLow	8.9	0.00	0.85	0.77	0.93	8.8	0.00	0.83	0.75	0.93
Easy vs HighHigh	7.1	0.00	0.79	0.74	0.88	6.9	0.00	0.76	0.66	0.87
Easy vs Difficult	13.6	0.00	0.93	0.90	0.96	14.3	0.00	0.93	0.88	0.97
LowLow vs Difficult	7.7	0.00	0.81	0.73	0.91	8.4	0.00	0.82	0.66	0.94
HighHigh vs Difficult	8.3	0.00	0.83	0.76	0.93	9.7	0.00	0.86	0.77	0.94
LowLow vs HighHigh	-0.2	0.87	-0.03	-0.43	0.33	0.6	0.59	0.10	-0.32	0.36
Reaction Time										
Easy vs LowLow	-3.4	0.00	-0.53	-0.73	-0.31	-6.5	0.00	-0.74	-0.92	-0.53
Easy vs HighHigh	-4.8	0.00	-0.66	-0.81	-0.56	-6.5	0.00	-0.74	-0.88	-0.61
Easy vs Difficult	-4.9	0.00	-0.67	-0.81	-0.53	-7.4	0.00	-0.78	-0.87	-0.71
LowLow vs Difficult	-3.3	0.00	-0.52	-0.75	-0.26	-6.0	0.00	-0.72	-0.84	-0.59
HighHigh vs Difficult	-1.9	0.08	-0.32	-0.56	-0.03	-4.6	0.00	-0.62	-0.78	-0.51
LowLow vs HighHigh	-2.4	0.03	-0.41	-0.64	-0.14	-4.0	0.00	-0.56	-0.75	-0.36
Efficiency										
Easy vs LowLow	-8.2	0.00	-0.83	-0.94	-0.74	-12.3	0.00	-0.90	-0.96	-0.84
Easy vs HighHigh	-6.2	0.00	-0.75	-0.88	-0.68	-7.5	0.00	-0.79	-0.91	-0.72
Easy vs Difficult	-8.5	0.00	-0.84	-0.92	-0.79	-11.9	0.00	-0.90	-0.94	-0.85
LowLow vs Difficult	-5.7	0.00	-0.72	-0.85	-0.61	-8.6	0.00	-0.83	-0.92	-0.73
HighHigh vs Difficult	-5.6	0.00	-0.72	-0.90	-0.59	-10.0	0.00	-0.86	-0.93	-0.80
LowLow vs HighHigh	-1.3	0.21	-0.23	-0.53	0.09	-2.4	0.03	-0.38	-0.55	-0.15
Performance Confidence										
Easy vs LowLow	3.2	0.01	0.50	0.19	0.81	6.7	0.00	0.75	0.66	0.89
Easy vs HighHigh	4.8	0.00	0.66	0.52	0.80	6.7	0.00	0.76	0.63	0.86
Easy vs Difficult	6.0	0.00	0.74	0.60	0.86	8.9	0.00	0.84	0.76	0.91
LowLow vs Difficult	4.6	0.00	0.64	0.53	0.77	5.3	0.00	0.67	0.55	0.81
HighHigh vs Difficult	3.9	0.00	0.58	0.36	0.79	5.4	0.00	0.68	0.51	0.85
LowLow vs HighHigh	2.4	0.03	0.40	0.13	0.64	2.4	0.03	0.38	0.08	0.64
Predicted Confidence										
Easy vs LowLow	2.1	0.05	0.37	0.04	0.68	3.9	0.00	0.56	0.36	0.73
Easy vs HighHigh	3.2	0.01	0.51	0.30	0.70	4.0	0.00	0.56	0.43	0.71
Easy vs Difficult	3.7	0.00	0.56	0.37	0.74	4.6	0.00	0.62	0.47	0.78
LowLow vs Difficult	3.8	0.00	0.57	0.41	0.74	3.6	0.00	0.53	0.36	0.69
HighHigh vs Difficult	2.2	0.05	0.37	0.05	0.71	1.7	0.10	0.29	0.01	0.52
LowLow vs HighHigh	2.9	0.01	0.46	0.21	0.68	1.3	0.20	0.22	-0.09	0.57

Supplementary Table 2: Error Awareness: Pairwise comparisons of participants' confidence judgments following errors vs correct responses.

	**Experiment 1** (df = 15); Block 1-6				**Experiment 2** (df = 17), Block 1-4 (pre-switch)			
	t	*p*	*r.e.*	CI	*t*	*p*	*r.e.*	CI
Easy	7.1	0.00	0.79	0.62, 0.91	6.1	0.00	0.72	0.61, 0.84
Low, low	5.4	0.00	0.70	0.57, 0.85	8.2	0.00	0.82	0.73, 0.91
High, high	6.3	0.00	0.75	0.60, 0.89	6.5	0.00	0.75	0.65, 0.85
Difficult	6.7	0.00	0.78	0.65, 0.90	4.3	0.00	0.60	0.42, 0.76

Supplementary Table 3: Average, min and max trial numbers per condition separately for both the CNV and both CPP analyses.

	**CNV**			**CPP**		
	Mean	Min	Max	Mean	Min	Max
Easy	94.6	58	119	74.4	51	88
Low, low	90.4	49	117	72.0	46	90
High, high	93.0	44	121	73.5	41	91
Difficult	91.1	55	120	71.6	50	90

Supplementary Notes:

Subject no 7.

As indicated in the Methods, staircasing failed for one participant. This participant achieved nearly identical performance in the easy condition (94.1% correct) as in the high mean, high variance condition (91.5% correct). At the same time, they scored similarly in the low mean, low variance (74.6% correct) and the difficult condition (74.4% correct).

Their performance confidence matched this behavior. Confidence was high in the easy condition (mean confidence: 5.72) and the high mean, high variance condition (mean confidence 5.76), while it was diminished in the low mean, low variance condition (mean confidence: 3.52) and the difficult condition (mean confidence 4.91). Finally, in line with the argument put forward based on the sample population data, this participant showed predicted confidence judgments that reflected their performance confidence more closely than their performance. They were similarly confident in the easy condition (mean confidence 5.78) and the high mean, high variance condition (mean confidence: 5.84). While their predicted confidence was largely decreased for the low mean, low variance condition (mean confidence: 3.03), their predicted confidence in the difficult condition was similar to their performance confidence in this condition (mean confidence: 4.97), which is unjustified considering mean accuracy, supporting the idea that performance confidence has a larger effect on predicted confidence than accuracy itself.

Supplementary Results:

Error Awareness

Participants' ratings of subjective confidence would not be interpretable if they were unable to veridically report on their errors above chance level. Therefore, for Experiment 1, we established that participants were indeed able to detect their errors and use the confidence scale accordingly. This was clearly the case, with average confidence scores of 3.4 following errors and of 4.7 following correct responses on our 6-point scale ($t(15) = 7.4$ $p < 0.001$ *r equivalent* = 0.80, 95% CI [0.65, 0.93]). This confidence difference between correct and incorrect responses was found to be significant in every condition (all *t*s > 5.4, all *p*s < 0.001, all *r equivalent* > 0.70, Supplement Table 2 for details). Participants were clearly able to detect their own errors (had good confidence resolution) and used the confidence scale in a meaningful way.

The same held for Experiment 2, where error awareness was assessed both in the first four experiment blocks with the original cue-condition contingencies, as well as in the two blocks after the switch of these contingencies. All these comparisons were found to be significant with confidence being higher after correct than incorrect responses in all four conditions, both in the original mapping blocks, as well as in the blocks after the switch (all $ts(17) > 4.3$, all *p*s < 0.001, all $\eta_p^2 > 0.60$, Supplemental Table 2 for details, all effects remain significant after Bonferroni correction).

Current Source Density analysis of the CPP

The CPP amplitude measure as described in the main text was our pre-planned analysis. However, the CPP has been previously reported to potentially be affected by additional components reflecting task difficulty (Kelly & O'Connell, 2013), which is why we also report an alternative analysis. In this previous work, it has been suggested that the condition-sensitivity of the amplitude may result from an overlay of the positive going CPP component, and fronto-central negative going components, associated with response preparation. This argument has been supported by the finding that amplitude differences in different conditions can be reduced by performing a current source density (CSD) correction during pre-processing (Kayser & Tenke, 2006). We therefore performed a CSD and reran all analyses. To summarize these extensive tests: The amplitude and slope differences between conditions remained the same way as in the presented non CSD-corrected data. In fact, in the majority of comparisons the effects of condition were slightly stronger in this analysis. If there was any systematic detectable difference, it would lie in the lack of an effect of posteriority on amplitude. This does not affect our main hypotheses.

<u>Slope</u>

1) Is there a general effect of condition on slope?

Slope showed a significant effect of CONDITION in the univariate ANOVA ($F(3,51) = 6.9$, $p < 0.001$,

η_p^2 = 0.29, 95% CI [0.17, 0.48]).

2) Is the effect of condition explained by fast responses?

A repeated-measures ANOVA with the factors RT (fast vs. slow) and CONDITION showed a significant effect of RT ($F(1,17) = 19.8$, $p < 0.001$, $\eta_p^2 = 0.53$, 95% CI [0.30, 0.78]) and in contrast to the non-CSD corrected analysis, the effect of CONDITION also reached significance ($F(3,51) = 4.1$, $p = 0.01$, $\eta_p^2 = 0.19$, 95% CI [0.1, 0.37]). There was no reliable interaction ($F(3,51) = 1.7$, $p = 0.18$, $\eta_p^2 = 0.08$, 95% CI [0.04, 0.26]).

3) Effect of condition in neutral trials

The univariate ANOVA testing the 4-level factor CONDITION revealed reliable effect of stimulus category, again in contrast to the non-CSD corrected analysis ($F(3,51) = 3.6$, $p = 0.03$, $\eta_p^2 = 0.34$, 95% CI [0.19, 0.59]). Again, we caution that because this analysis relies on small trial numbers, we would not over-confidently interpret this effect.

4) Condition x Switch

As in the main analysis, we found a significant effect of CONDITION ($F(3,51) = 9.5$, $p < 0.001$, $\eta_p^2 = 0.35$, 95% CI [0.22, 0.53]) and no significant effect of SWITCH ($F < 1$). and no reliable interaction ($F < 1$). Note that this analysis does not control for the effects of RT.

As in the analysis presented in the main text, these results provide solid evidence for a modulation of CPP slope by the same factors that also affect RTs. There is no evidence for an effect of cued confidence on evidence accumulation as reflected in CPP slope.

<u>*Amplitude*</u>

1) *Condition x Posteriority*

   A repeated measures ANOVA with factors of CONDITION (4 levels) and POSTERIORITY (3 levels; 1: CZ, 2: CPZ, 3: PZ) revealed a main effect of CONDITION ($F(3,51) = 4.2$, $p = 0.01$, $\eta_p^2 = 0.19$, 95% CI [0. 07, 0.40]) but no main effect of POSTERIORITY ($F(2,34) = 2.6$, $p = 0.08$, $\eta_p^2 = 0.13$, 95% CI [0.02, 0.41]). There is also no reliable interaction ($F(6,102) = 1.3$, $p = 0.25$, $\eta_p^2 = 0.07$, 95% CI [0.04, 0.22). Note that the null-effect for posteriority is a deviation from the main analysis, which however, does not affect the interpretation of the results, which rely on the main effect of condition.

2) *T-tests between amplitudes by condition*

   None of the direct comparisons between neighboring conditions reaches statistical significance (all *p*s > 0.12).

3) *Is the effect of condition explained by differences in RT?*

Trials sorted into slow and fast by median split were entered into a 4x2 repeated measures ANOVA, yielding a significant main effect of CONDITION ($F(3,51) = 4.3$, $p = 0.008$, $\eta_p^2 = 0.2$, 95% CI [0.11, 0.41], and no main effect of RT ($F(1,17) = 3.3$, $p = 0.07$, $\eta_p^2 = 0.17$, 95% CI [0.007, 0.51]), with no interaction ($F(3,136) = 1.1$, $p = 0.31$, $\eta_p^2 = 0.06$, 95% CI [0.03, 0.22]. There is a small difference to the main analysis in that RT doesn't reach significance, however, the main result of an effect of CONDITION remains.

4) *Conditions x posteriority x switch*

The factors CONDITION (4 levels) and POSTERIORITY (3 levels; 1: CZ, 2: CPZ, 3: PZ) and SWITCH (pre-switch/switch; that is Blocks 4 and 5) revealed a main effect of CONDITION ($F(3,51) = 5.0$, $p = 0.003$, $\eta_p^2 = 0.23$, 95% CI [0.03, 0.38]) no main effect of POSTERIORITY ($F(2,34) = 2.1$, $p = 0.14$, $\eta_p^2 = 0.11$, 95% CI [0.00, 0.29]), and no main effect of SWITCH ($F < 1$). Again, contrary to our hypothesis, there was no significant interaction between CONDITION and SWITCH ($F(3,51) = 2.3$, $p = 0.08$, $\eta_p^2 = 0.12$, 95% CI [0.00, 0.26]),. None of the other interactions were significant (Fs < 1.4, ps > 0.2).

5) *Conditions x posteriority x switch for easy and difficult*

In contrast to the main analysis, the exploratory test for an interaction between the effect of condition and switch when only easy and difficult trials are considered did not reach significance ($F(1,17) = 1.2$, $p = 0.28$, $\eta_p^2 = 0.07$, 95% CI [0.00, 0.33]). In fact, this repeated measures ANOVA yielded only one main effect, that of CONDITION ($F(1,17) = 8.6$, $p = 0.01$, $\eta_p^2 = 0.34$, 95% CI [0.02, 0.58]).

6) *Condition for neutral cues*

Only analyzing the trials that were not preceded by predictive cues, but all cued with the same non-informative cue, the univariate ANOVA testing the 4-level factor CONDITION showed the expected main effect ($F(3,51) = 4.5$, $p = 0.007$, $\eta_p^2 = 0.21$, 95% CI [0.13, 0.38]). Pairwise comparisons of neighboring conditions show no significant differences (all p>0.1)

Drift-diffusion model

Previous modeling of the same task has shown that participants adjust their response criteria (decision threshold) within each condition (Boldt, 2015). Because of the close link between CPP amplitude and decision threshold (O'Connell et al., 2012), we modeled behavioral data across both experiments in a drift diffusion model. Data from Experiment 1 and the pre-switch blocks of Experiment 2 were combined and submitted to the *EZdiff* Matlab function, part of The Diffusion Model Analysis Toolbox (DMAT; Wagenmakers, van der Maas, & Grasman, 2007). The resulting four condition-specific values for boundary separation (the variable which corresponds to decision threshold in the DDM) were entered in to a repeated measures ANOVA with the four level factor condition, which resulted in a significant effect ($F(3,99) = 44.7$, $p < 0.001$, $\eta_p^2 = 0.58$, 95% CI [0.44, 0.72]): As previously shown, and in accordance with the CPP results, we found the highest value for the easy condition ($M = 0.12$, $SEM = 0.002$), followed by the low mean, low variance condition ($M = 0.11$, $SEM = 0.002$), the high

mean, high variance condition (*M* = 0.11, *SEM* = 0.002), and finally the difficult condition (*M* = 0.10, *SEM* = 0.001; Suppl. Figure 1a). The same pattern was found for drift-rate, that is the measure of evidence accumulation (easy: *M* = 0.22, *SEM* = 0.01; low mean, low variance: *M* = 0.14, SEM = 0.01; high mean, high variance: *M* = 0.14, *SEM* = 0.01; difficult: *M* = 0.08, SEM = 0.01; Suppl. Figure 1b), and the repeated measures ANOVA likewise yielded a significant effect of condition ($F(3,99) = 120.3$, $p < 0.001$, $\eta_p^2 = 0.78$, 95% CI [0.75, 0.83]).

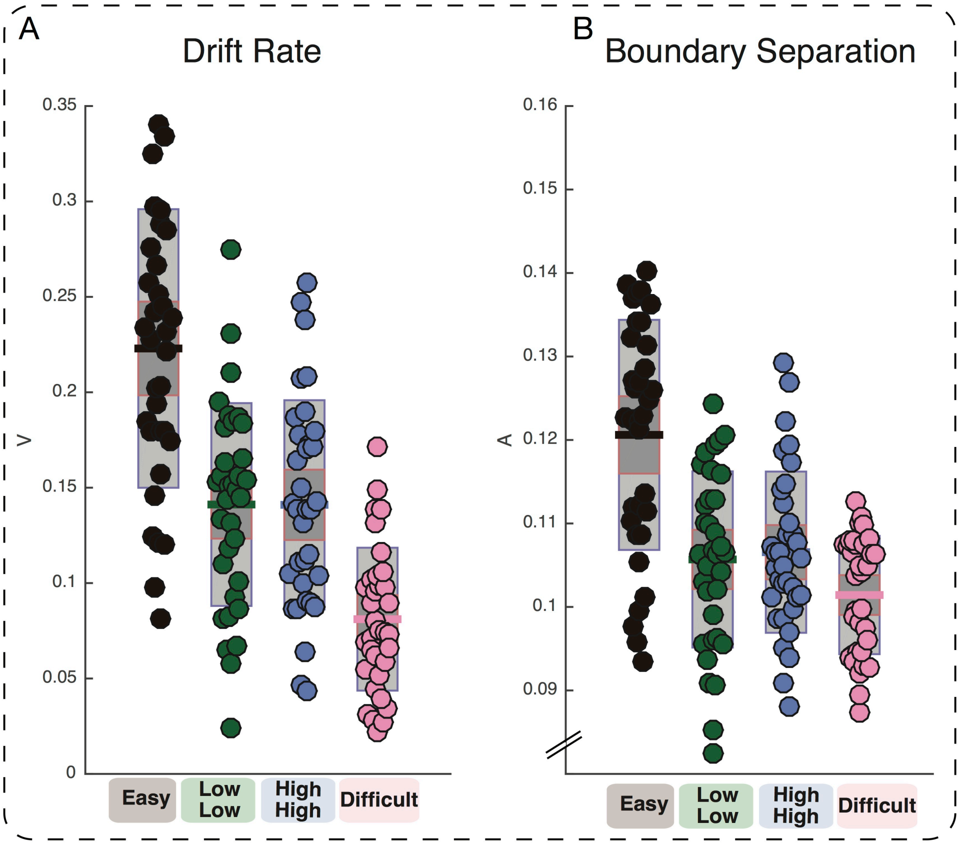

Supplement Figure 1: Modeled Drift rate (left) and Boundary Separation (right) both show strong modulation across conditions. Both are particularly elevated for easy trials, showing a comparable pattern to the CPP amplitude across both fast and slow trials, in line with the proposal that the CPP reflects a decision-to-bound threshold.

Supplementary References:

Boldt, A. (2015). *Metacognition in decision making* (Doctoral dissertation). Retrieved from https://ora.ox.ac.uk:443/objects/uuid:5d9b2036-cc42-4515-b40e-97bb3ddb1d78

Kayser, J., Tenke, C.E. (2006). Principal components analysis of Laplacian waveforms as a generic method for identifying ERP generator patterns: I. Evaluation with auditory oddball tasks. *Clinical Neurophysiology*, *117*(2), 348-368. doi:10.1016/j.clinph.2005.08.034

Kelly, S. P., & O'Connell, R. G. (2013). Internal and external influences on the rate of sensory evidence accumulation in the human brain. *The Journal of Neuroscience : The Official Journal of the Society for Neuroscience*, *33*(50), 19434–19441. doi:10.1523/JNEUROSCI.3355-13.2013

O'Connell, R. G., Dockree, P. M., & Kelly, S. P. (2012). A supramodal accumulation-to-bound signal that determines perceptual decisions in humans. *Nature Neuroscience*, *15*(12). doi:10.1038/nn.3248

Wagenmakers, E. J., Van Der Maas, H. L., & Grasman, R. P. (2007). An EZ-diffusion model for response time and accuracy. *Psychonomic bulletin & review*, *14*(1), 3-22.
